# Impact of mystical belief and traditional healing on the health-seeking practice of conversion disorder in middle- and low-income countries

**DOI:** 10.1097/MS9.0000000000000109

**Published:** 2023-02-17

**Authors:** Anusha Sumbal, Mirza M.A. Baig, Ramish Sumbal

**Affiliations:** Dow University of Health Sciences, Karachi, Pakistan

**Keywords:** conversion disorder, mystical belief, socioeconomic status

## Abstract

Conversion disorder is a somatoform condition in which patients present with a range of neurologic deficits and sensorimotor loss with no obvious pathology. There has been a rising trend in the incidence of conversion disease in countries with low socioeconomic backgrounds, accounting to be one-third of ambulatory visits in middle and low-income countries (MLIC). However, even with such a high prevalence health-seeking practice for conversion disorder is low in MLIC. One possible reason for such behavior could be the high prevalence of mystical beliefs and traditional healing in MLIC. Existing economic distress with limited healthcare resources convinces people to opt for traditional and local healers who make use of mystical and superstition beliefs prevalent in those regions to offer prospering and cheaper methods of treatment. In this scenario, addressing and counseling mythological fallacies and the use of an economically friendly ‘holistic model’ of treatment should be adopted in these countries.

Impact of mystical belief and traditional healing on the health-seeking practice for conversion disorder in middle- and low-income countries (MLICs).

Functional neurological symptom disorder, popularly known as conversion disorder is a type of somatoform disorder in which a patient presents with severe motor and sensory symptoms such as paralysis, tremor, and seizure^1^. Since this is a psychiatric illness and not any neuropathology, even on detailed radiologic imaging and an extensive blood workup, there is no conclusive finding^1^. There has been an increasing trend in the incidence of conversion disorder in MLICs over the past couple of decades^2^. It accounts for the second-leading cause of neurology consults and about one-third of new patients in ambulatory emergency visits are cases of conversion disorder patients^2^. Especially in countries with low socioeconomic status, conversion disorder is becoming increasingly endemic. In Monroe County, United States spikes of 854 new patients were reported in just a time of 5 years^2^. Similarly, in South East Asia, conversion disorder stands for 12.4% of admission in Psychiatric wards^1^.

Even with such a high prevalence of conversion disorder, the health-seeking behavior by the family and patients of conversion illness in MLICs is very poor^2^. The reporting ratio of patients with symptoms of conversion in these regions is as low as 0.2%, and the psychiatric consultation rate for diagnosed conversion disorder patients is less than 5%^2^. Such a decrease in health-seeking behavior could be due to many reasons, such as misconception, wrong or delayed diagnosis, poor counseling, and a lack of documented evidence for ‘medically unexplained symptoms’ in conversion disease^3^. One such reason includes the high prevalence of mystical belief and traditional healing, particularly in low- to middle-income countries^4^. In MLICs, the use of a biomedical model to treat mental illness is way too limited, whereas the use of traditional and spiritual healing is greater than 70%^4^. Such a profound establishment of mystical belief and superstition has been a consequence of illiteracy, limited healthcare standards, and a high poverty rate^5^. In MLICs, the chain of healthcare and infrastructure are weak, medical assistance is expensive, and the threshold of inflation is far beyond reach^6^. Here the role of traditional healers comes into play^6^. These traditional local healers make use of the fear and financial limitations of these populations to make them believe that a mystical or supernatural force is causing these ‘unexplained symptoms’ in them^5^. They also integrate elements of cultural and religious ideologies to support their superstition, promising to get rid of their pain and illness^6^.

Taking into account the financial crisis, it is quite convincing to the people of low- and middle-income countries (MLIC) to choose this cheaper and easier-to-access method of healing rather than opting for a proper healthcare consultation and medical assistance, which is relatively expensive for them^5^. Also to mention that the costly treatment regime and follow-up fees significantly contribute to the hesitation toward medical assistance^6^. The role of superstition in MLICs is so strong that its account to be a key factor in health care decisions and that up to 80% population of MLIC it is considered completely normal to seek traditional healing for common diseases^5^. These methods are cheap, convincing, and culturally significant thus, approaching local faith healers and use of medicinal herbs is economically and socially easier for them, thus, patients and families of conversion illness do not even show up to healthcare and are reluctant to use expensive pharmacologic agents available which seems unsatisfactory to them^4^.

According to the literature, 73% of patients with conversion disorder and their families approached local faith healers as the first line of treatment^7^. Whereas, 33% used traditional remedies, herbs, and hypnosis to treat their patients^7^. It was quite unfortunate that seeking healthcare support is not the foremost choice of people, possibly because these countries have quite limited access to a modern medicinal framework which outcasts their ability to understand the pathophysiology of these and easily fabricate it to already existing mythologies and paranormal beliefs^4^. The mindset of these subjects behind their neglecting psychiatric help is that around one-third of them firmly believe that a ‘supernatural’ force is causing these symptoms^7^. A French neurologist, Hippolyte Bernheim, pointed out that it was not until the 19th century that conversion disorder was considered hysterical; before this, it was popularly considered a ‘demonic possession’^8^. Therefore, this popular mystical belief in MLICs convinces the common people that evil spirits and metaphysical forces are the causative agents^4^. That is why about 27% of patients who used amulets (sacred stones) found a cure, and a significant group used herbs and home remedies rather than using the correct medicines and psychiatric support available^4^,^7^.

Now, this is a medical challenge for MLICs. Increasing conversion disorder patients and decreasing health-seeking approaches are an emerging threat to the future healthcare system in the regions. In this entire scenario, we need a multipronged approach to integrate possible control over these fallacies, myths, and mystical beliefs^9^. Taking into account the financial status of patients’ limited use of anticonvulsive and antidepressive medicine and the dominant use of cognitive behavioral therapy, mindfulness, and psychodynamic therapies should be preferred^3^,^9^. These therapies are relatively inexpensive and easy to communicate, thereby increasing patient compliance and adherence to treatment^9^. Also, the use of older labels such as ‘pseudoseizure’ or ‘false/hysterical paralysis’ creates a negative energy of self-blame and guilt in patients, we encourage psychiatrists to use terms like ‘converted’ or ‘somatization’ which reduce stigmatization and minimizes psychological distress in the patients^3^,^9^. Even rather than directly refuting patient cultural values and mythical beliefs, integrating cultural, and social beliefs with psychological help to create a ‘holistic model’ of treatment should be adopted^9^. Valuing a patient’s traditional and financial background helps to establish a bond of trust and respect between healthcare and patients^3^,^4^. Nevertheless, financial aid and the dispersion of modern health facilities in these countries would create a better long-term perspective result^4^. Hence, we believe that the high prevalence of mystical belief in MLICs could be a potential barrier to a proper understating and health-seeking practice of conversion disorder.

## Ethical approval

Not applicable.

## Consent

Not applicable.

## Sources of funding

None to declare.

## Author contribution

A.S., M.M.A.B., and R.S.: concept and writing the paper.

## Conflicts of interest disclosure

None to declare

## Research registration unique identifying number (UIN)

1. Name of the registry: not applicable.

2. Unique identifying number or registration ID: not applicable.

3. Hyperlink to your specific registration (must be publicly accessible and will be checked): not applicable.

## Guarantor

None to declare.

## Provenance and peer review

Not commissioned, externally peer-reviewed.
